# SDF-1/CXCR4-Mediated Stem Cell Mobilization Involved in Cardioprotective Effects of Electroacupuncture on Mouse with Myocardial Infarction

**DOI:** 10.1155/2022/4455183

**Published:** 2022-08-09

**Authors:** Tian-tian Zhao, Jia-jia Liu, Jing Zhu, Han Li, Ya-chao Wang, Yue Zhao, Shui-jin Shao, Hai-dong Guo, Fang-fang Mou

**Affiliations:** ^1^Department of Anatomy, School of Basic Medicine, Shanghai University of Traditional Chinese Medicine, Shanghai 201203, China; ^2^Academy of Integrative Medicine, Shanghai University of Traditional Chinese Medicine, Shanghai 201203, China

## Abstract

Stem cell-based therapeutic strategies have obtained a significant breakthrough in the treatment of cardiovascular diseases, particularly in myocardial infarction (MI). Nevertheless, limited retention and poor migration of stem cells are still problems for stem cell therapeutic development. Hence, there is an urgent need to develop new strategies that can mobilize stem cells to infarcted myocardial tissues effectively. Electroacupuncture (EA) intervention can improve cardiac function and alleviate myocardial injury after MI, but its molecular mechanism is still unclear. This study is aimed at observing the effects of EA treatment on the stem cell mobilization and revealing possible mechanisms in the MI model of mice. EA treatment at Neiguan (PC6) and Xinshu (BL15) acupoints was conducted on the second day after the ligation surgery. Then, the number of stem cells in peripheral blood after EA in MI mice and their cardiac function, infarct size, and collagen deposition was observed. We found that the number of CD34-, CD117-, Sca-1-, and CD90-positive cells increased at 6 h and declined at 24 h after EA intervention in the blood of MI mice. The expression of CXC chemokine receptor-4 (CXCR4) protein was upregulated at 6 h after EA treatment, while the ratio of LC3B II/I or p-ERK/ERK showed a reverse trend. In addition, there was obvious difference in EF and FS between wild-type mice and CXCR4^+/−^ mice. The infarct size, collagen deposition, and apoptosis of the injured myocardium in CXCR4^+/−^ mice increased but could be ameliorated by EA. In a word, our study demonstrates that EA alleviates myocardial injury via stem cell mobilization which may be regulated by the SDF-1/CXCR4 axis.

## 1. Introduction

Myocardial infarction (MI), as a noninfectious cardiovascular disease, is triggered by acute or persistent ischemia and hypoxia of the coronary artery [1], which brings heavy economic burden to society and families [2]. Subsequent cardiomyocyte necrosis, myocardial fibrosis, and ventricular remodeling aggravate MI. Thrombolysis, angioplasty, electronic implants, and coronary artery bypass surgery have been considered to be timely and effective in the treatment of MI clinically. These approaches can only rescue the viable cardiomyocytes in the marginal zone of the infarction instead of addressing the loss of cardiomyocytes [3]. Currently, stem cell-based therapy has been declared as a promising approach for cardiac regeneration [4]. Stem cells can mediate immune regulation, induce proliferation, and facilitate renovation of injury due to their unique properties of paracrine, homing, and multidirectional differentiation, which offers a tremendous hope to numerous patients [5]. According to their different evolution stages, stem cells are classified into embryonic stem cells and adult stem cells [1]. Among them, most researchers focus on bone marrow-derived stem cells (bMSCs), which are one of the adult stem cells. BMSCs reside in bone marrow normally. They will be mobilized into the bloodstream and homed to the sites of myocardial injury and take part in tissue repair during ischemia or hypoxia. Granulocyte colony-stimulating factor (G-CSF) is used as a mobilization agent for mobilizing endogenous stem cells and is now undergoing phase III trials. However, it has disadvantages such as high cost and apparently long-term side effects [6]. Moreover, the curative effect of stem cell-based therapy is limited and cannot achieve the anticipated goals owing to different cell delivery routes, low engraftment, and low retention rate. It has been proven that 11% adult stem/progenitor cells retain in the myocardium of intramyocardial injection while only 2% retain after intravenous injection [7, 8]. Given those inevitable limitations, mobilizing more endogenous stem cells to the damaged sites plays a critical role in enhancing the therapeutic efficacy following MI.

The stromal cell-derived factor (SDF)-1/CXCR4 is essential in the migration and homing of stem cells [9]. SDF-1, also known as CXCL12, is the principal specific ligand of CXCR4. It is upregulated significantly in the organism suffering from inflammation, ischemia, hypoxia, and angiogenesis, which indicates that SDF-1 might be necessary for tissue repair and regeneration [10, 11]. A great deal of studies have been done to support that SDF-1 can recruit various stem cells and chemokines to the injured myocardium, thereby participating in myocardial injury repair following MI [12]. Furthermore, the downstream cellular signals of CXCR4 will also be activated, affecting recruitment, proliferation, migration, and apoptosis in injured sites [13]. Electroacupuncture (EA), with a low-voltage electrical current stimulation in specific acupoints, has been demonstrated to play a unique role in the prevention and treatment of cardiovascular diseases [14]. EA has been widely used to treat MI due to their simplicity, efficiency, cheapness, and practicality [15]. It has been reported that EA at PC6 acupoint may improve cardiac function in isoproterenol-induced hypertrophy mice [16, 17]. Zeng et al. reported that EA at bilateral PC6 acupoint could alleviate myocardial damage and improve prognosis via the AMPK-mTOR signaling pathway [18]. Furthermore, EA can also promote the survival of exogenous bMSCs after transplantation and then improve the cardiac function after MI [19]. Another research reveals that EA can even facilitate endogenous MSC mobilization into the peripheral blood under normal physiological conditions [20]. However, there are still no reports about whether EA promotes endogenous bMSC migration and homing to the injured myocardium.

Thus, PC6 and BL15 acupoints have been selected in line with meridian theory. The effect of EA on stem cell mobilization and SDF-1/CXCR4 signaling pathway after MI has been observed. These results may provide a theoretical foundation for further exploring the underlying mechanisms of EA treatment.

## 2. Materials and Methods

### 2.1. Experimental Animals

Adult male C57BL/6 (B.W., 20-25 g) and CXCR4 heterozygous (CXCR4^+/−^) mice were raised in the animal experiment center of Shanghai University of Traditional Chinese Medicine. They were maintained at 26 ± 2°C with free water and food. All experimental procedures involved in this study were authorized by the animal ethics committee of Shanghai University of TCM and the Animal Research Committee of Shanghai (no. PZSHUTCM190628003). Efforts were made to reduce the number of mice and improve the utilization rate of mice as far as possible during the whole procedure.

### 2.2. Myocardial Infarction Model

MI mice were established by ligature of the left anterior descending branch under isoflurane anesthetization [21]. Briefly, each mouse was fixed in a supine position, and then, endotracheal intubation was used, followed by ventilation using a rodent respirator (Harvard Apparatus, USA). After cardiac exposure, the left anterior descending coronary artery was ligated with surgical sutures followed by thoracic incision suture. Then, penicillin was administrated intramuscularly in MI mice on the second day after modeling.

### 2.3. Groups and EA Treatments

EA treatment was conducted on the second day after operation. Acupoints on PC6 and BL15 was defined based on previous studies [16, 22, 23]. The needles, 15 mm in length, were inserted vertically into 3 mm depth of corresponding acupoints with twirling, lifting, and thrusting and then were connected to an electroacupuncture therapeutic instrument (KWD-808I, Yingdi, Changzhou, China) for 30 min once a day.

Firstly, to observe the effects of EA on stem cell mobilization after MI, 16 mice were randomly classified into 2 groups (*n* = 8 in each group): (i) MI group and (ii) EA group. Mice in the EA group were given EA intervention once after MI. Half of the animals in each group were sacrificed at 6 h and the other half at 24 h after MI.

Secondly, to analyze the phenotype of CXCR4^+/−^ mice, 1-month-old heterozygous mice, the wild-type mice in the same cage were applied for experiments. There were 4 mice in the WT group and CXCR4^+/−^ group. All animals were directly sacrificed without treatment.

Thirdly, to further investigate the heterozygote and wild-type mice' response to MI, 32 mice were randomly divided into 8 groups (*n* = 4 in each group): (i) 1 M group, (ii) 2 M group, (iii) 3 M group, and (iv) 5 M group. Each group was further divided into WT and CXCR4^+/−^ groups. All mice were subjected to echocardiography at 1 day and 7 days after MI.

Finally, to explore the role of the SDF-1/CXCR4 axis in stem cell mobilization induced by EA after MI, 3-month-old mice were divided into 3 groups (*n* = 6 for each group): (i) MI+CXCR4^+/−^ group, (ii) EA+CXCR4^+/−^ group, and (iii) EA+WT group. All mice were subjected to MI modeling while the mice in the EA+CXCR4^+/−^ group and EA+WT group were given EA treatment for 5 days after MI.

### 2.4. Tissue Processing for Histology

Blood was collected through abdominal aorta and then conserved into a centrifugal tube containing anticoagulant. Subsequently, heart tissues were isolated rapidly and sliced into two parts through the infarct core. One part of those tissues was frozen quickly by using liquid nitrogen and then stored at -80°C for the following western blot analysis. The other part was fixed with 4% paraformaldehyde, dehydrated with sucrose, and embedded in Tissue-Tek OCT (Sakura, USA) in turn. Finally, 10 *μ*m slices were prepared under freezing microtome (Thermo, USA) and stored at -20°C for Masson staining and HE staining.

### 2.5. Flow Cytometry Analysis

The peripheral blood was initially dissociated by Red Blood Cell Lysis Buffer to remove erythrocytes. The cell suspension was washed twice with cold 0.01 M PBS and then incubated with an FITC-labeled CD34 monoclonal antibody (Thermo, USA)and CD117 monoclonal antibody (Thermo, USA) for 90 minutes and CD90 monoclonal antibody (Thermo, USA), Sca-1 monoclonal antibody (Thermo, USA), and CXCR4 monoclonal antibody (Thermo, USA) for 30 minutes at 4°C, respectively. The samples were detected with a Cytoflex flow cytometer, and the data were analyzed with TreeStar Flowjo software.

### 2.6. Western Blot Analysis

In brief, heart tissues were homogenized in a RIPA lysis buffer containing protease and phosphatase inhibitors (Beyotime Biotechnology, Shanghai, China). The protein samples were extracted by ultracentrifugation at 4°C. And the protein concentrations were detected by using a BCA Protein Assay Kit. Equal amounts (40 *μ*g) of denatured protein samples were separated by appropriate sodium dodecyl sulfate polyacrylamide gel electrophoresis (SDS-PAGE) and transferred to the polyvinylidene fluoride (PVDF) membranes (Millipore, United States). To prevent nonspecific protein binding sites from binding to antibodies, the membranes were cultured in 5% nonfat milk. The membranes were then incubated with Rabbit anti-p-ERK1/2 (1 : 1000, Cell Signaling Technology), rabbit anti-ERK1/2 (1 : 1000, Cell Signaling Technology), rabbit anti-Beclin 1 (1 : 1000, Cell Signaling Technology), rabbit anti-GAPDH (1 : 1000, Cell Signaling Technology), rabbit anti-LC3B (1 : 1000, Abcam), anti-collagen III (1 : 1000, Abcam), and anti-Col1A1 (1 : 1000, Cell Signaling Technology), respectively, overnight at 4°C. Subsequently, the membranes were incubated with the corresponding secondary antibody at room temperature for 2 h. The membranes of protein samples were observed with an enhanced chemiluminescence (Pierce Biotechnology, Waltham, MA, USA) and visualized by a gel image analysis system (Bio-Rad).

### 2.7. Echocardiography

M-mode echocardiography was detested by using a high-resolution in vivo ultrasound microimaging system (Vevo Visualsonics 2100; Visualsonics, Toronto, ON, Canada) on the 1st and 7th days after the ligation surgery. Briefly, all mice were anesthetized by inhaling isoflurane and fixed in supine position to provide the left parasternal short-axis view. Left ventricular end-systolic volume (LVESV), left ventricular end-diastolic volume (LVEDV), left ventricular end-diastolic diameters (LVDd), and left ventricular end-systolic diameters (LVDs) were obtained. Ejection fraction (EF) and fractional shortening (FS) were calculated by using the following formulas: EF (%) = (LVEDV − LVESV)/LVEDV × 100 and FS (%) = (LVDd − LVDs)/LVDd × 100.

### 2.8. HE Staining and Masson's Staining

According to a previously published literature, HE staining and Masson's staining were performed [24]. The slices were kept at room temperature for 10 minutes and then washed with 0.01 M PBS. According to the manufacturers' recommended protocol, those slices were stained with relevant reagents. For the calculation of the infarction size, the sum of the infarcted epicardial perimeter plus the infarcted endocardial perimeter was divided by the sum of the left ventricular epicardial perimeter and the left ventricular endocardial perimeter, and the data were expressed in percentage.

### 2.9. Statistical Analysis

The data were expressed with the mean ± standard error of the mean (SEM). ANOVA and *t*-test were conducted to analyze the difference in this study via Prism 7.0. The *p* value was less than 0.05, which meant that the data of two groups showed statistical difference.

## 3. Results

### 3.1. Effect of EA on the Mobilization of Bone Marrow Stem Cells after MI

**Figure 1:**
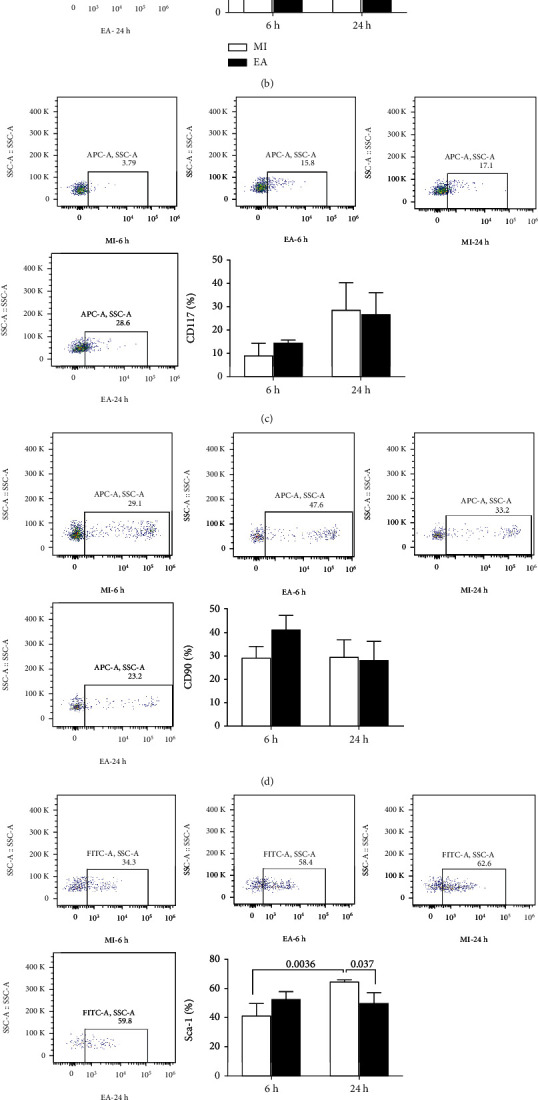
Effect of EA on bone marrow stem cell mobilization in peripheral blood of mice at 6 h and 24 h following MI. (a) Timeline for the treatment and detection. (b) The number of CD34-positive stem cells. (c) The number of CD117-positive stem cells. (d) The number of CD90-positive stem cells. (e) The number of Sca-1-positive stem cells. (f) The number of CXCR4-positive stem cells (*n* = 4).

EA intervention could improve cardiac function impaired by MI though regulating inflammatory factors and chemokines. To further observe the effects of EA on the mobilization of bMSCs after MI, the numbers of CD34-, CD117-, CD90-, Sca-1-, and CXCR4-positive cells in peripheral blood were detected via flow cytometric analysis (Figure 1(a)). The survival rate of mice was 80% at 6 h and 70% at 24 h. The number of peripheral CD34-positive cells increased at 6 h in the MI group (*p* = 0.0024, Figure 1(b)). The number of CD117- or CD90-positive cells displayed similar changes but without statistical difference (*p* > 0.05, Figures 1(c) and 1(d)). Moreover, the number of peripheral Sca-1-positive cells increased in the MI group at 24 h compared with those at 6 h after MI (*p* = 0.0036, Figure 1(e)). The number of peripheral Sca-1-positive cells decreased in the EA group in comparison with those in the MI group (*p* = 0.037, Figure 1(e)). There was no significant difference of CXCR4-positive cell number between the EA and MI groups (*p* > 0.05, Figure 1(f)).

### 3.2. Effects of EA on the Expression of LC3B, p-ERK and CXCR4 Protein Levels in Myocardial Tissue after MI

**Figure 2:**
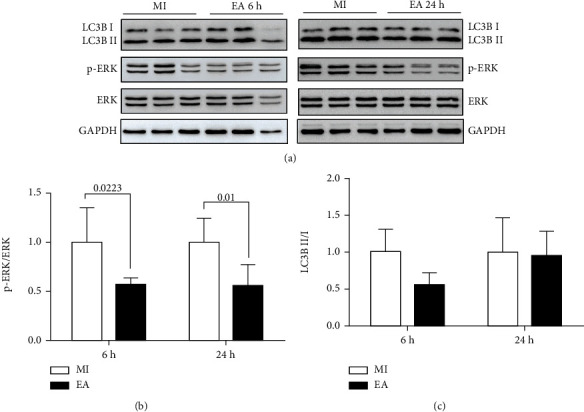
Effect of EA on the expression of p-ERK/ERK and LC3B II/I in myocardial tissue at 6 h and 24 h following MI. (a) The protein expression levels of LC3B, p-ERK, and ERK at 6 h and 24 h following MI were detected by western blot. (b, c) The semiquantitative data of western blots for LC3BII/I and p-ERK/ERK (*n* = 4).

Next, myocardium LC3B and p-ERK protein levels were detected among groups. We found that the myocardium p-ERK/ERK ratio was lower in the EA group at 6 h (*p* = 0.0233) and 24 h (*p* = 0.01) in comparison with that in the MI group after MI (Figures 2(a) and 2(b)). There was no difference of LC3B II/I between MI and EA groups (*p* > 0.05, Figures 2(a) and 2(c)).

**Figure 3:**
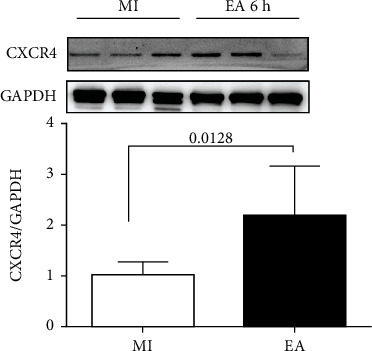
Effect of EA on the expression of CXCR4 in myocardial tissue at 6 h following MI. The CXCR4 protein bands of the myocardium at 6 h following MI were detected by western blot, and the semiquantitative data of western blots for CXCR4 was utilized (*n* = 4).

Myocardium CXCR4 protein levels were higher in the EA group compared with those in the MI group at 6 h after MI (*p* = 0.0128, Figure 3). These results indicated that CXCR4 and downstream ERK signal might play a critical role in the mobilization of bone marrow stem cells mediated by EA after MI.

### 3.3. Cardiac Functions, and the Expression of CXCR4 and Apoptosis and Autophagy Related Proteins in C57BL/6 CXCR4^+/−^ Mice

**Figure 4:**
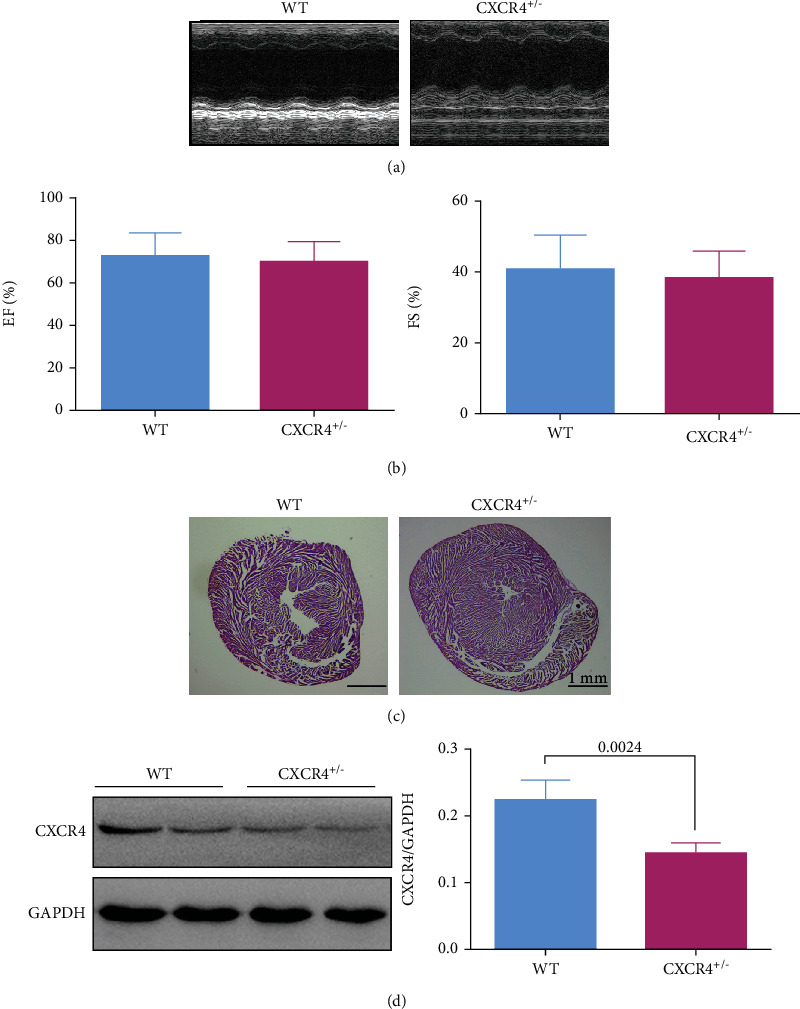
The characterization of CXCR4^+/−^ mice. (a) The cardiac function of mice was detected by echocardiography. (b) The parameters of EF and FS were compared between the two groups. (c) HE staining was performed to evaluate the cardiac structure. (d) The expression of CXCR4 protein was detected by western blot, and the semiquantitative data of western blot for CXCR4 were shown (*n* = 4).

SDF-1 and CXCR4 deletions impaired the cardiovascular system and hematopoietic function [11]. In our study, CXCR4 heterozygous (CXCR4^+/−^) mouse was established for determining whether CXCR4 could affect cardiac function in MI. Hence, 1-month-old heterozygous mice and the corresponding wild-type mice from the same cage were raised for echocardiography and HE staining. No difference was found in EF and FS between WT and CXCR4^+/−^ mice (*p* > 0.05, Figures 4(a) and 4(b)). Histological analysis also showed that the myocardial cells in CXCR4^+/−^ mice remained well arranged, and there was no difference in cardiac structure between these two groups (*p* > 0.05, Figure 4(c)). The above results suggested that the cardiac function of CXCR4 ^+/−^ mice was similar to that of wild-type mice without MI.

In addition, the expression of CXCR4 protein in myocardial tissue of these mice was quantified by WB. In comparison with WT mice, the expression of CXCR4 protein in the CXCR4 ^+/−^ mouse myocardium was lower significantly (*p* = 0.0024, Figure 4(d)).

**Figure 5:**
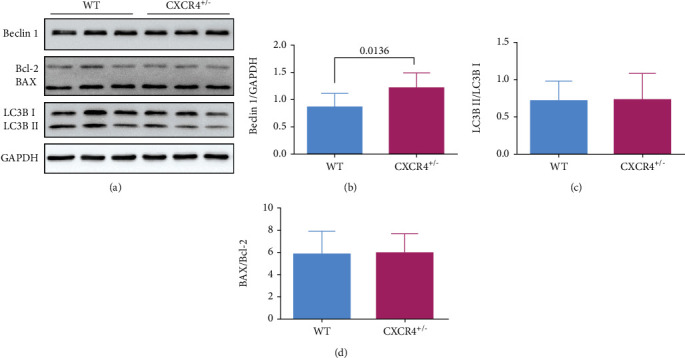
Effect of CXCR4 deficiency on the expression of BAX, Bcl-2, Beclin1, and LC3B proteins under physiological conditions. (a) The expressions of BAX, Bcl-2, Beclin1, and LC3B were detected by western blot. (b–d) The semiquantitative data of western blots for Beclin1, LC3B II/I, and BAX/Bcl-2 (*n* = 4).

Besides, there was no difference in the ratio of BAX/Bcl-2 and LC3B II/I between those two groups (*p* > 0.05, Figure 5).

### 3.4. Cardiac Function in C57BL/6 CXCR4^+/−^ Mice after MI

**Figure 6:**
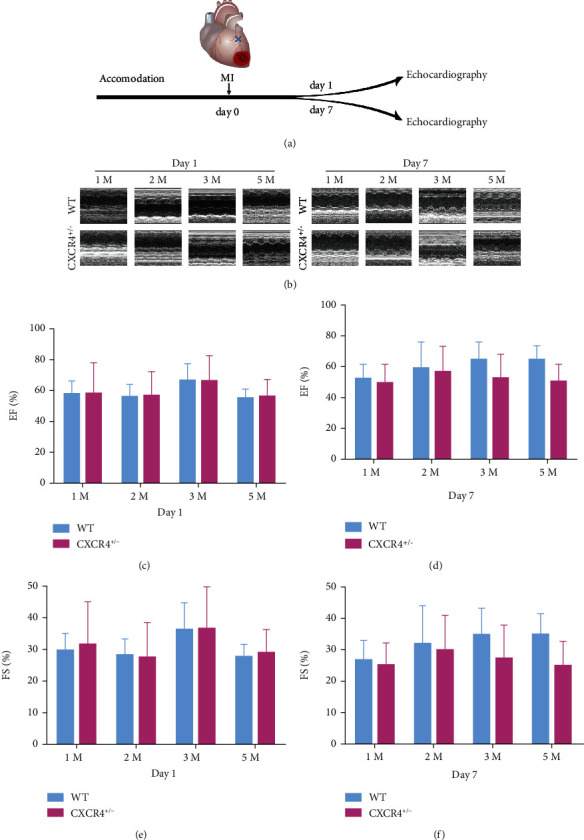
Effect of different ages on cardiac function in WT and CXCR4^+/−^ groups after MI. (a) Timeline for the treatment and detection. (b) The representative echocardiography images. The parameters of EF (c) and FS (e) on the 1st day after MI were compared between WT and CXCR4^+/−^ groups. The parameters of EF (d) and FS (f) on the 7th day after MI were compared between WT and CXCR4^+/−^ groups (*n* = 4).

Thus, 1-, 2-, 3-, and 5-month CXCR4 ^+/−^ mice and corresponding WT mice from the same cage were subjected to MI, and echocardiography was performed on the 1st day and the 7th day after MI (Figure 6(a) and 6(b)). The survival rate of mice was 70% at day 1 and 75% at day 7. There was no difference in EF and FS between CXCR4 ^+/−^ mice and corresponding WT mice (*p* > 0.05, Figures 6(c)–6(f)).

### 3.5. Effect of EA on Infarct Size and Collagen Content Cardiac Function in C57BL/6 CXCR4^+/−^ Mice after MI

**Figure 7:**
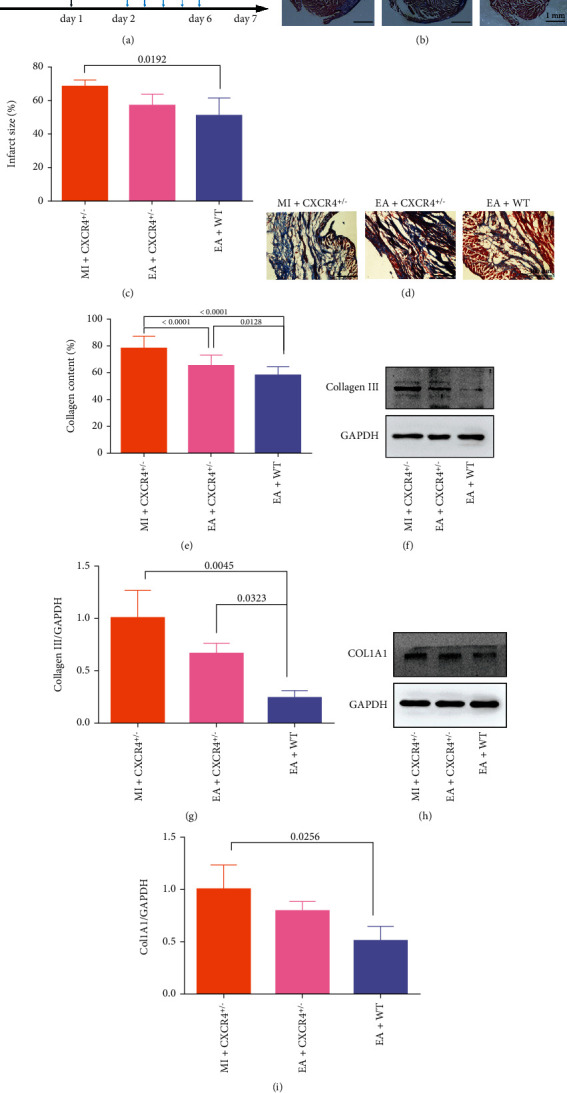
Effect of EA on infarct size and collagen content in the infarcted area after MI. (a) Timeline for the treatment and detection. (b, d) Masson's trichrome staining was performed to detect the infarction size and collagen content in each group. Scale bar = 100 *μ*m. (c) The infarct size was expressed as the ratio of (infarct area/the whole cross‐sectional area) × 100%. (e) The collagen content was expressed as the ratio of (fibrotic tissue area/the whole myocardial surface area) × 100% (*n* = 6). (f, h) The expressions of collagen III and Col1A1 were detected by western blot. (g, i) The semiquantitative data of western blots for collagen III and Col1A1 (*n* = 6).

Next, the infarct size and collagen deposition in the infarct region were assessed by Masson trichrome staining (Figure 7(a)). The survival rate of mice was 75%. The myocardium of CXCR4^+/−^ mice appeared to have the biggest infarct size when they suffered from MI. The infarct size of the EA+WT group was lower in comparison with that in the MI+CXCR4 ^+/−^ group (*p* = 0.0192, Figures 7(b) and 7(c)). Compared with the MI+CXCR4^+/−^ group, collagen content was lower in the EA+CXCR4 ^+/−^ group and EA+WT group (*p* < 0.0001, Figures 7(d) and 7(e)). Moreover, collagen content in the EA+WT group decreased in comparison with that in the EA+CXCR4 ^+/−^ group (*p* = 0.0128, Figures 7(d) and 7(e)). In addition, to further analyze the collagen content, the expressions of collagen III and Col1A1 were detected. Compared with the MI+CXCR4^+/−^ group and EA+CXCR4^+/−^ group, the expressions of collagen III in the EA+WT group were significantly decreased (*p* = 0.0045 and *p* = 0.0323, Figures 7(f) and 7(g)). The level of Col1A1 in the EA+WT group was obviously lower than that in the MI+CXCR4^+/−^ group (*p* = 0.0256, Figures 7(h) and 7(i)).

### 3.6. Effect of EA on Myocardial Apoptosis in C57BL/6 CXCR4^+/−^ Mice after MI

**Figure 8:**
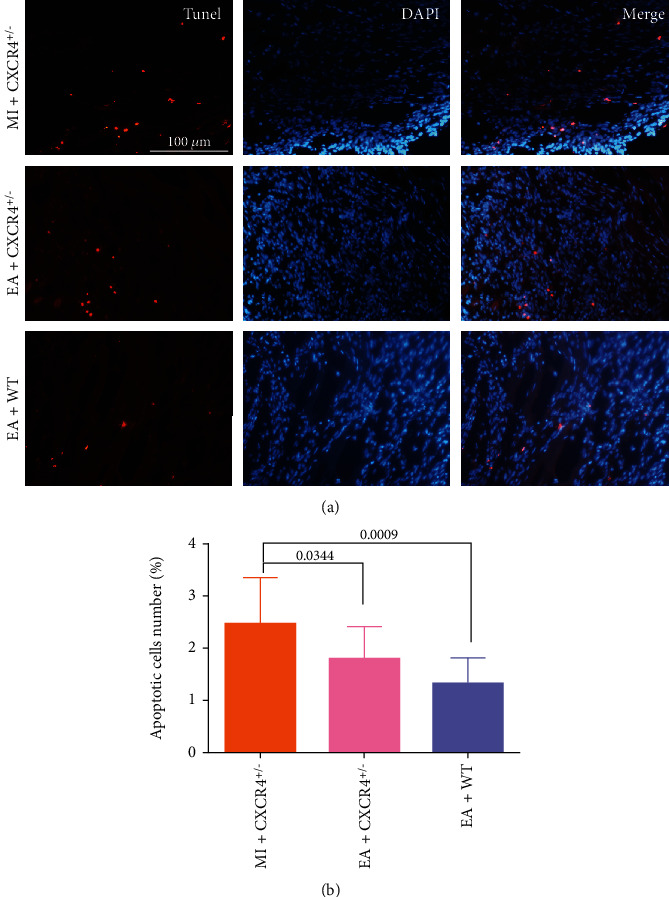
Effect of EA on myocardial apoptosis in the marginal zone of infarction after MI. (a) TUNEL staining was used to detect cell apoptosis. Scale bar = 100 *μ*m. (b) The number of positive apoptotic cells was quantified with percentage (*n* = 6).

Apoptosis in the marginal zone of infarction was assessed by TUNEL staining. As it is shown in Figure 8, a large amount of positive apoptotic cells are observed in the MI+CXCR4^+/−^ group and EA+CXCR4^+/−^ group while the number of positive apoptotic cells is less in the EA+WT group compared with the MI+CXCR4^+/−^ group (*p* = 0.0009) and EA+CXCR4^+/−^ group (*p* = 0.0344, Figures 8(a) and 8(b)). These results further proved that CXCR4 might play an important role in the repair of cardiac function induced by EA after acute myocardial infarction.

### 3.7. Effect of EA on the level of p-ERK, CXCR4 and SDF-1 in C57BL/6 CXCR4^+/−^ Mice after MI

**Figure 9:**
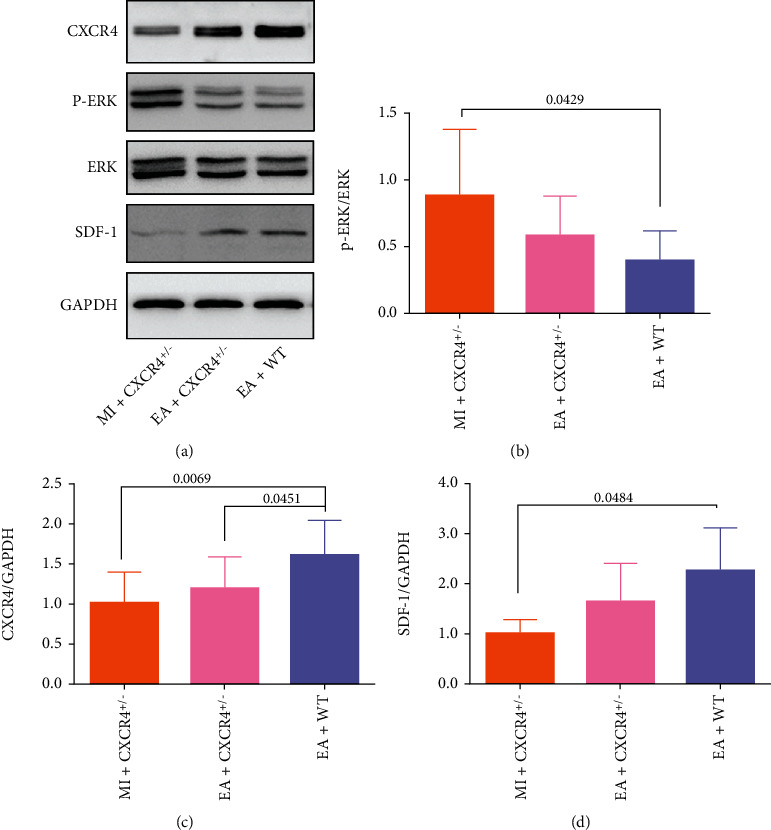
Effect of EA on the expression of p-ERK, ERK, CXCR4, and SDF-1 proteins in myocardial tissue after MI. (a) The protein expression levels of p-ERK, ERK, CXCR4, and SDF-1 among groups. (b–d) The semiquantitative data of western blots for p-ERK/ERK CXCR4 and SDF-1 (*n* = 6).

To further investigate the potential molecular mechanisms underlying mobilization induced by EA after MI, the expressions of p-ERK, ERK, CXCR4, and SDF-1 proteins were assessed. Compared with the MI+CXCR4^+/−^ group, the p-ERK/ERK ratio was lower in the EA+WT group (*p* = 0.0429, Figures 9(a) and 9(b)). In comparison with the MI+CXCR4^+/−^ (*p* = 0.0069) and EA+CXCR4^+/−^ groups (*p* = 0.0451), CXCR4 protein expression was higher in the EA+WT group (Figures 9(a) and 9(c)). Compared with the MI+CXCR4^+/−^ group, SDF-1 protein expression was higher in the EA+WT group (*p* = 0.0484, Figures 9(a) and 9(d)). These results suggested that SDF-1, bound to CXCR4, might inhibit its downstream ERK signal pathway and improve mobilization of bone marrow stem cells after MI mediated by EA.

## 4. Discussion

EA, modified by an ancient acupuncture therapy, has emerged as a critical alternative strategy in the treatment of various diseases. Notably, many experimental studies have demonstrated that EA treatment ameliorates cardiac function following MI as it can mediate autophagy [25], oxidative stress [26], and inflammatory reaction [27] and improve cardiomyocyte apoptosis post-MI [28]. In addition, Salazar et al. reported that MSCs were promoted to leave their niche and enter the blood circulation following EA intervention at specific points [29]. EA can also facilitate the transplanted neural stem cells migration to the injured site [30]. In this study, the number of blood CD34-, CD117-, Sca-1-, and CD90-positive cells is observed after EA treatment in MI mice, for evaluating EA effects in the mobilization of stem cells after MI. CD34-positive cells have already been confirmed to be beneficial for the repair in various animal models of MI [31, 32]. Although c-kit-positive cells are incapable of differentiating into cardiomyocytes, direct intramyocardial administration of them can effectively improve cardiac function after MI [33]. It has been found that homed Sca-1-positive cells exert cardioprotection in lethally irradiated old mice who have received young Sca-1 cells and then suffer from MI [34]. The mobilization of stem cells contributes to alleviating myocardial injury, and EA treatment may contribute to the mobilization.

The SDF-1/CXCR4 axis plays a key role in mobilizing stem cells to the sites of myocardial injury [35]. Under steady-state conditions, stem cells are anchored to the endosteal osteoblasts by the combination of SDF-1 and CXCR4 [36]. Only when the interaction between SDF-1 and CXCR4 is cleaved or SDF-1 is degraded or CXCR4 expression is inhibited by its antagonist (e.g., AMD3100) in the microenvironment of bone marrow can stem cells be delivered to peripheral blood [37, 38]. It has been reported that higher level of SDF-1 is detected and persists for 7 days in the myocardial tissues after MI [36]. Abundant evidences have proven that high levels of SDF-1 and CXCR4 located in the injured site may participate in regulating mobilization, homing, angiogenesis, embryonic development, immune response, and so on [12, 39]. Despite the fact that our flow cytometry analysis showed that the counts of CXCR4-positive cells in peripheral blood displayed no substantial difference between two groups both at 6 h and at 24 h post-EA treatment, the level of CXCR4 protein in the EA group was higher than that in the MI group at 6 h in this study. In addition, we previously identified that the expression of SDF-1 protein in myocardial tissue was upregulated significantly at the 7th day following EA treatment. These results imply that EA treatment may protect the heart from ischemia via prolonging the expression of SDF-1 and CXCR4 and ensuring the temporal alignment of peaks. Considering its significance of mobilization, we propose a hypothesis that SDF-1/CXCR4 may be implicated in the mobilization of stem cells induced by EA treatment after MI. To verify our hypothesis, CXCR4 heterozygous mice, appearing to have normal cardiac structure and function, were applied to the experimental study. We found that infarct size, collagen deposition, and apoptosis of CXCR4^+/−^ mice were much worse than those of normal mice but could be ameliorated by EA treatment. The data show that CXCR4 has a notable influence on the cardioprotective action mediated by EA and may be involved in the mobilization of stem cells induced by EA after MI.

Apart from its direct potency of mobilization and migration, SDF-1 is also involved in cardiac repair via multiple signaling pathways [36]. SDF-1 binds to its G protein-coupled receptor CXCR4, which activates G*α*i protein and further initiates its downstream signaling cascades, including mitogen-activated protein kinase (MAPK) and phosphoinositide 3 kinase (PI3K) [12, 36]. ERK is a member of the MAPK family. The ERK signal transduction pathway, as one of the MAPK cascaded in eukaryotic cells, is extensively reported to regulate cell growth, development, and differentiation [40, 41]. There is growing evidence that ERK plays a substantial role in cardiovascular disease, including cardiac hypertrophy, myocardial infarction, atherosclerosis, and vascular restenosis [42]. Recently, ERK signaling pathway inhibition is suggested as one of the possible mechanisms mediating D-limonene's cardioprotection in the myocardial injury model [43]. Furthermore, a study reveals that EA at PC6 acupoint for 14 days can improve cardiac function by reducing the phosphorylation levels of ERK in the left ventricular myocardial hypertrophy model [44]. In our studies, the ratio of p-ERK/ERK in the myocardial tissue drops obviously at 6 h or 24 h after EA treatment, suggesting that cardioprotection of EA is associated with inhibition of the ERK signal transduction pathway. In order to verify whether the ERK signaling pathway is regulated by CXCR4 in this condition, the expressions of p-ERK and ERK proteins were assessed in CXCR4^+/−^ mice who subjected to MI as well as EA treatment. The results showed that this ratio was higher in the EA+CXCR4^+/−^ group than in the EA+WT group, but without statistical significance. Given few samples and individual differences, we will increase the sample size and make the data more persuasive later.

In addition, we also measured the expression of autophagy-related protein LC3B in myocardial tissue. We found that the LC3B II/I ratio was downgraded in the EA group and had a negative correlation with the expression of CXCR4 at 6 h after EA treatment. Our results indicated that autophagy might be another mechanism of cardioprotection mediated by EA following MI and CXCR4 might be an upstream activator of LC3B. Recently, an increasing number of studies suggest that EA can preserve cardiac function by regulating various classical autophagy pathways [18, 25, 45, 46]. Therefore, the effect of autophagy cannot be ignored. We also found that the protein expression of LC3B did not change and remained at normal levels in CXCR4^+/−^ mice, which means that LC3B is not regulated by CXCR4. Nevertheless, the reduced CXCR4 could cause higher level of Beclin1 that was thought to regulate autophagy by forming complexes with different proteins. Accumulated documents demonstrate that the interaction between Beclin1 and Bcl-2 plays an essential role in the crosstalk between autophagy and apoptosis [47, 48]. Thus, the upregulated Beclin1 does not mean the initiation of Beclin1-mediated autophagy. We assume that CXCR4 is not involved in the regulation of autophagy. Further verification still needs to be done under certain pathological conditions.

In conclusion, our study shows that EA at PC6 and Xinshu acupoints improves cardiac function and promotes stem cell mobilization after MI. Upregulated expression of SDF-1 and CXCR4 as well as decreased expression of p-ERK and the autophagy protein LC3B after EA treatment suggest that its underlying molecular mechanism may be associated with SDF-1/CXCR4/ERK axis-mediated mobilization and autophagy. We also find that reduced CXCR4 can partially reverse EA-induced cardioprotection. However, the role of the SDF-1/CXCR4 axis in mobilization mediated by EA treatment still requires further verification. This study provides a new perspective for the research of EA treatment in MI.

## Data Availability

The data that support the findings of this study are available from the corresponding author upon reasonable request.

## Supplementary Materials

Supplementary Materials Figure S1: gene identification of CXCR4 heterozygous mice with agar gelatin electrophoresis (note: nos. 2, 3, 4, 5, 6, and 7 are CXCR4^+/-^ mice; nos. 1, 8, and 9 are wild-type mice).
