# Supplementary material for: SDF-1/CXCR4-Mediated Stem Cell Mobilization Involved in Cardioprotective Effects of Electroacupuncture on Mouse with Myocardial Infarction
Source: Oxid Med Cell Longev. 2022 Aug 9;2022:4455183. doi: 10.1155/2022/4455183 (PMC9381195; doi:10.1155/2022/4455183)
Supplement: Supplementary Materials — Figure S1: gene identification of CXCR4 heterozygous mice with agar gelatin electrophoresis (note: nos. 2, 3, 4, 5, 6, and 7 are CXCR4+/- mice; nos. 1, 8, and 9 are wild-type mice). [file 4455183.f1.docx]

**Supplementary Information**


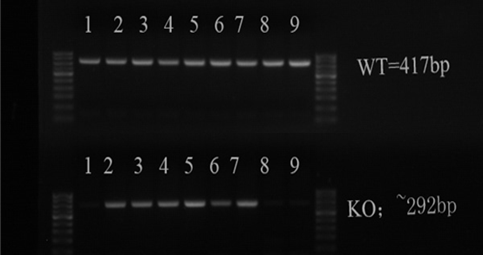


Figure S1. Gene identification of CXCR4 heterozygous mice with agar gelatin electrophoresis (Note: No.2, 3, 4, 5, 6, 7 are CXCR4 + /- mice; No.1, 8, 9 are wild-type mice).
